# Possible Contribution of Inflammation-Associated Hypoxia to Increased K_2P_5.1 K^+^ Channel Expression in CD4^+^ T Cells of the Mouse Model for Inflammatory Bowel Disease

**DOI:** 10.3390/ijms21010038

**Published:** 2019-12-19

**Authors:** Kyoko Endo, Hiroaki Kito, Ryo Tanaka, Junko Kajikuri, Satoshi Tanaka, Elghareeb E. Elboray, Takayoshi Suzuki, Susumu Ohya

**Affiliations:** 1 Department of Pharmacology, Division of Pathological Sciences, Kyoto Pharmaceutical University, Kyoto 607-8414, Japan; kd16001@poppy.kyoto-phu.ac.jp (K.E.); rt.hk0304@gmail.com (R.T.); tanaka-s@mb.kyoto-phu.ac.jp (S.T.); 2 Department of Pharmacology, Graduate School of Medical Sciences, Nagoya City University, Nagoya 467-8601, Japan; kito@med.nagoya-cu.ac.jp (H.K.); kajikuri@med.nagoya-cu.ac.jp (J.K.); 3 Department of Complex Molecular Chemistry, The Institute of Scientific and Industrial Research, Osaka University, Osaka 567-0047, Japan; elborayvip@yahoo.com (E.E.E.); tkyssuzuki@sanken.osaka-u.ac.jp (T.S.); 4 Faculty of Science, South Valley University, Qena 83523, Egypt

**Keywords:** K^+^ channel, K_2P_5.1, inflammatory bowel disease, CD4^+^ T cell, hypoxia, HIF-1α

## Abstract

Previous studies have reported the up-regulation of the two-pore domain K^+^ channel K_2P_5.1 in the CD4^+^ T cells of patients with multiple sclerosis (MS) and rheumatoid arthritis (RA), as well as in a mouse model of inflammatory bowel disease (IBD). However, the mechanisms underlying this up-regulation remain unclear. Inflammation-associated hypoxia is involved in the pathogenesis of autoimmune diseases, such as IBD, MS, and RA, and T cells are exposed to a hypoxic environment during their recruitment from inflamed tissues to secondary lymphoid tissues. We herein investigated whether inflammation-associated hypoxia is attributable to the increased expression and activity of K_2P_5.1 in the splenic CD4^+^ T cells of chemically-induced IBD model mice. Significant increases in hypoxia-inducible factor (HIF)-1α transcripts and proteins were found in the splenic CD4^+^ T cells of the IBD model. In the activated splenic CD4^+^ T cells, hypoxia (1.5% O_2_) increased K_2P_5.1 expression and activity, whereas a treatment with the HIF inhibitor FM19G11 but not the selective HIF-2 inhibitor exerted the opposite effect. Hypoxia-exposed K_2P_5.1 up-regulation was also detected in stimulated thymocytes and the mouse T-cell line. The class III histone deacetylase sirtuin-1 (SIRT1) is a downstream molecule of HIF-1α signaling. We examined the effects of the SIRT1 inhibitor NCO-01 on K_2P_5.1 transcription in activated CD4^+^ T cells, and we found no significant effects on the K_2P_5.1 transcription. No acute compensatory responses of K_2P_3.1–K_2P_5.1 up-regulation were found in the CD4^+^ T cells of the IBD model and the hypoxia-exposed T cells. Collectively, these results suggest a mechanism for K_2P_5.1 up-regulation via HIF-1 in the CD4^+^ T cells of the IBD model.

## 1. Introduction

Alkaline pH-activated K^+^ channels (K_2P_5.1, 16.1, 17.1) belonging to the two-pore domain K^+^ (K_2P_) channel superfamily contribute to setting the resting potential and control of Ca^2+^ signaling, and they have also been implicated in inflammation and cancer development [1,2,3]. K_2P_5.1 (also referred to TASK-2 and KCNK5) plays an important role in cell volume regulation, renal bicarbonate reabsorption, and cancer cell proliferation [4,5,6]. K_2P_5.1 expression and activity is up-regulated in the CD4^+^ T cells of patients with autoimmune diseases such as rheumatoid arthritis (RA) [7], and multiple sclerosis (MS) [8] and those from inflammatory bowel disease (IBD) model mice [9]; however, the mechanisms that underly inflammatory response-mediated K_2P_5.1 up-regulation in CD4^+^ T cells remain unclear.

The dysregulated transcriptional, translational, and post-translational expression of K^+^ channels is associated with the pathogenesis of immune and inflammatory disorders [10]. Three major K^+^ channel subtypes—voltage-gated K_V_1.3, Ca^2+^-activated K_Ca_3.1, and K_2P_5.1—have been extensively studied in T cells [7,8,11,12]. We recently reported the histone deacetylase (HDAC)-mediated up-regulation of K_Ca_3.1 in the CD4^+^ T cells of IBD model mice [13,14]. However, no significant changes were observed in the expression levels of K_2P_5.1 transcripts following a treatment with selective class I HDAC inhibitors, suggesting that the mechanisms responsible for K_Ca_3.1 and K_2P_5.1 transcription in T cells under inflammatory conditions differ.

Hypoxia is linked to brain inflammation in MS patients [15], synovial inflammation in RA patients [16], and intestinal inflammation in IBD patients [17]. Hypoxia-inducible factors (HIFs) are heterodimeric transcriptional factors that consist of an oxygen-labile α subunit and a constitutively stable β subunit and that are involved in innate and adaptive immune activation. T cells are exposed to hypoxic conditions during their recruitment from inflamed tissues to secondary lymphoid tissues, such as the spleen and mesenteric lymph nodes, and HIF-1α is strongly expressed in T cells that infiltrate the inflamed mucosa in IBD patients [18]. HIF-1α plays a prominent role in the pathogenesis of inflammatory diseases by promoting inflammatory gene expression [19]. The leukocyte and myeloid cell-specific knockdown of HIF-1α has been found to result in more severe colonic inflammation with increased levels of pro-inflammatory cytokines in dextran sulfate sodium (DSS)-induced IBD model mice [18,20,21,22], indicating that hypoxia underlies the polarization of inflammatory T cells such as type 1 T helper (Th1) and Th17 cells in inflamed tissues. The hypoxic microenvironment is responsible for the control of Ca^2+^ homeostasis through altered K^+^ channel expression and function. For example, chronic hypoxia was reported to post-transcriptionally decrease the functional activity of the voltage-gated K^+^ channel K_V_1.3 by preventing forward trafficking from the trans-Golgi to the plasma membrane in T cells [23]. In contrast, an increase in the activity of K_2P_5.1 was found to be induced under sustained hypoxia in B cells, and K_2P_5.1 was found to be transcriptionally up-regulated via an HIF-1α-mediated signaling pathway [24]. Yuan et al. reported that hypoxia enhanced K_2P_5.1 protein expression in the carotid body [25].

Hypoxia-induced decreases in nicotinamide adenine dinucleotide (NAD^+^) were shown to reduce the activity of the class III NAD^+^-dependent HDAC, and sirtuin-1 (SIRT1) [26]. Furthermore, SIRT1 was found negatively regulate inflammatory pathways such as signal transducer and activator of transcription-3 (STAT3), SMAD7, and nuclear factor (NF)-κB [27], and it was also found to be down-regulated in IBD patients and IBD model mice [28]. However, SIRT1-mediated modifications to inflammation-associated ion channels have not yet been examined in detail.

In the present study, in order to elucidate the mechanisms that underly the increased K_2P_5.1 activity in the inflammatory CD4^+^ T cells of IBD model mice, we focused on the transcriptional regulation of K_2P_5.1 by inflammation-associated hypoxia.

## 2. Results

### 2.1. Up-Regulation of HIF-1α in Splenic CD4^+^ T Cells of DSS-Induced IBD Model Mice

**Figure 1:**
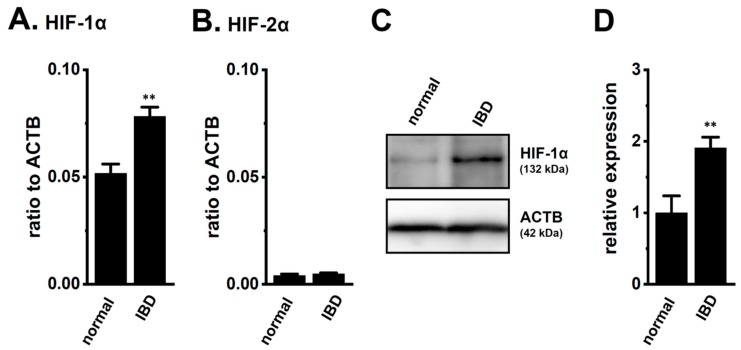
Increased expression of hypoxia-inducible factor (HIF)-1α in the splenic CD4^+^ T cells of dextran sulfate sodium (DSS)-induced inflammatory bowel disease (IBD) model mice. (**A**,**B**) Real-time PCR assay for HIF-1α (**A**) and -2α (**B**) in the splenic CD4^+^CD25^−^ T cells of ‘normal’ and ‘IBD’ model mice (*n* = 4). Expression levels are shown as a ratio to β-actin (ACTB). (**C**,**D**) HIF-1α protein expression (132 kDa) in the splenic CD4^+^ T cells of ‘normal’ and ‘IBD’ model mice. Protein lysates of the examined cells were probed by immunoblotting with anti-HIF-1α (upper panel) and anti-ACTB (42 kDa, lower panel) antibodies on the same filter (**C**). Summarized results were obtained as the optical density of HIF-1α and ACTB band signals (**D**). After compensation for the optical density of the HIF-1α protein band signal with that of the ACTB signal, the HIF-1α signal in ‘normal’ mice was expressed as 1.0 (*n* = 4). Results are expressed as means ± SEM. **: *p* < 0.01 vs. normal mice (normal).

We assessed the expression levels of HIF-1α in the splenic CD4^+^ T cells of normal and IBD model mice. As shown in our previous study [9], the expression levels of K_2P_5.1 and interferon (IFN)-γ transcripts in CD4^+^ T cells were higher in IBD model mice than in normal mice (*n* = 4 mice for each group, *p* = 0.0000 and *p* = 0.0002 for K_2P_5.1 and IFN-γ, respectively) (Supplementary Figure S1A,B). As shown in Figure 1A, the expression levels of HIF-1α transcripts in splenic CD4^+^CD25^−^ T cells were approximately 50% higher in the IBD model mice than in the normal mice (*n* = 4, *p* = 0.0052). HIF-2α transcripts were less abundantly expressed in the CD4^+^CD25^−^ T cells of both groups, and no significant differences were found between the groups (*n* = 4, *p* = 0.4439) (Figure 1B). Immunoblots of HIF-1α were performed with whole lysates of the CD4^+^ T cells. A band with a molecular weight of approximately 130 kDa that reacted with the anti-HIF-1α antibody was observed in both groups (Figure 1C). Similar to previous studies [17,18,19,21,22], the summarized results showed that the protein expression levels of HIF-1α in the CD4^+^ T cells were significantly higher in the IBD model mice than in the normal mice (*n* = 4, *p* = 0.0083) (Figure 1D). These results suggest that the CD4^+^ T cells of the IBD model were exposed to hypoxic conditions during their recruitment from the inflamed colon to the spleen, resulting in HIF-1α being strongly expressed in inflammatory T cells.

### 2.2. Enhancement of K_2P_5.1 Transcription by the Exposure to Hypoxia (1.5% O_2_) in Stimulated Splenic CD4^+^ T Cells of Mice

**Figure 2:**
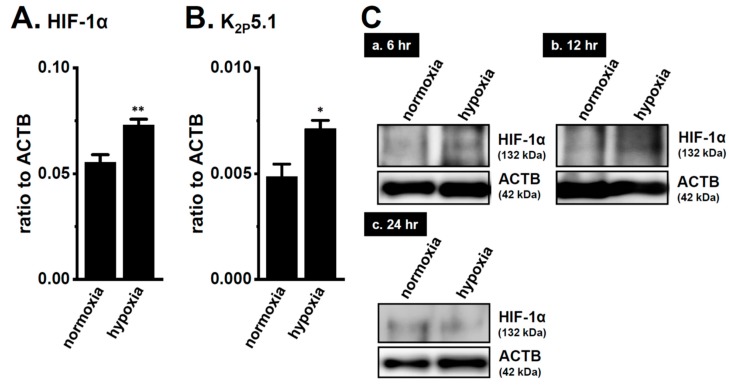
Up-regulation of the two-pore domain K^+^ channel (K_2P_)5.1 by hypoxia for 12 h in the concanavalin-A (Con-A)-stimulated splenic CD4^+^ T cells of mice. Real-time PCR assay for HIF-1α (**A**) and K_2P_5.1 (**B**) in splenic CD4^+^ T cells exposed to hypoxic conditions (1.5% O_2_) (*n* = 4 mice). Expression levels are shown as a ratio to ACTB. (**C**) HIF-1α protein expression (132 kDa) in splenic CD4^+^ T cells exposed to hypoxic conditions for 6 (**a**), 12 (**b**), and 24 (**c**) h. Protein lysates of the examined cells were probed by immunoblotting with anti-HIF-1α (upper panel) and anti-ACTB (42, kDa, lower panel) antibodies on the same filter. Results are expressed as means ± SEM. *, **: *p* < 0.05, 0.01 vs. normoxia.

We recently demonstrated the up-regulation of K_2P_5.1 with an increase in HIF-1α expression in mouse splenic CD4^+^ T cells stimulated by concanavalin-A (Con-A) for 24–48 h [29]. Twenty-four hours after stimulation by Con-A, Con-A-stimulated CD4^+^ T cells were exposed to hypoxia (1.5% O_2_) for an additional 24 h. The expression levels of K_2P_5.1 transcripts were approximately 50% higher in the hypoxia-exposed CD4^+^ T cells (*p* = 0.0246) (Figure 2B) with the HIF-1α up-regulation (*p* = 0.0077) (Figure 2A) than in those exposed to normoxia (20.8% O_2_) (*n* = 4). Immunoblots of HIF-1α were then obtained by using stimulated CD4^+^ T cells that were exposed to normoxia and hypoxia for 24 h. No significant differences were observed in the HIF-1α-specific band signals (132 kDa) between both groups (Figure 2(Cc)). On the other hand, the stronger expression of the HIF-1α proteins was noted in the hypoxia group exposed for 6 and 12 h (Figure 2(Ca,b)). It has been reported that HIF-1α is continuously active even though HIF-1α proteins are suppressed by long-term hypoxia [19]. In our previous study [9], immunoblots of K_2P_5.1 were successfully performed by using a rabbit polyclonal anti-K_2P_5.1 antibody that was purchased from Santa Cruz Biotechnology; however, this antibody has since been discontinued. In the present study, we examined immunoblots of K_2P_5.1 by using other commercially-available anti-K_2P_5.1 antibodies under several experimental conditions; however, no signals of the K_2P_5.1 proteins at approximately 45 kDa (predicted molecular weight) for the analysis of CD4^+^ T cell lysates in both groups were detected, and the detected band signals with different molecular weights were not disappeared by the preincubation with excess antigens.

**Figure 3:**
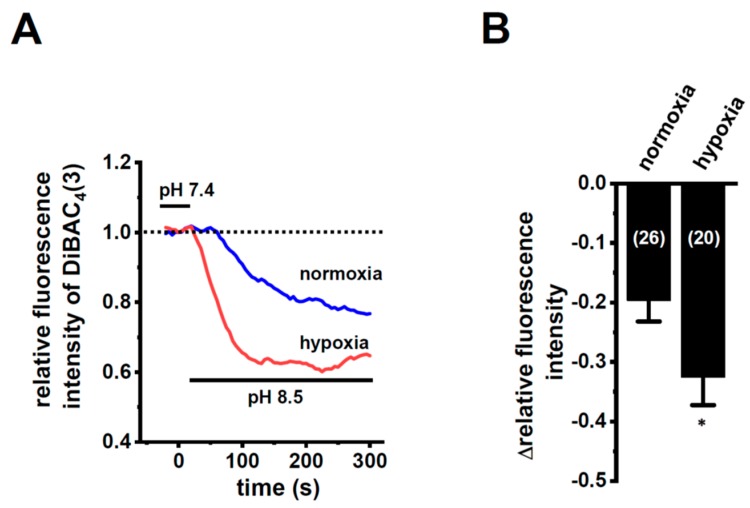
Enhanced K_2P_5.1 activity by hypoxia for 24 h in stimulated splenic CD4^+^ T cells of mice. (**A**) Time course of voltage-sensitive fluorescent dye imaging of alkaline-pH (pH 8.5)-induced hyperpolarizing responses in splenic CD4^+^ T cells. The fluorescent intensity of DiBAC_4_(3) before the change in pH from 7.4 to 8.5 (at 0 s) is expressed as 1.0. Images were measured every 5 s. (**B**) Summarized results of alkaline pH-induced hyperpolarizing responses. Cells were isolated from four different mice in each group. Cell numbers used in experiments are shown in parentheses. The values for fluorescent intensity were obtained by measuring the average for 1 min (12 images). Results are expressed as means ± SEM. *: *p* < 0.05 vs. normoxia.

**Figure 4:**
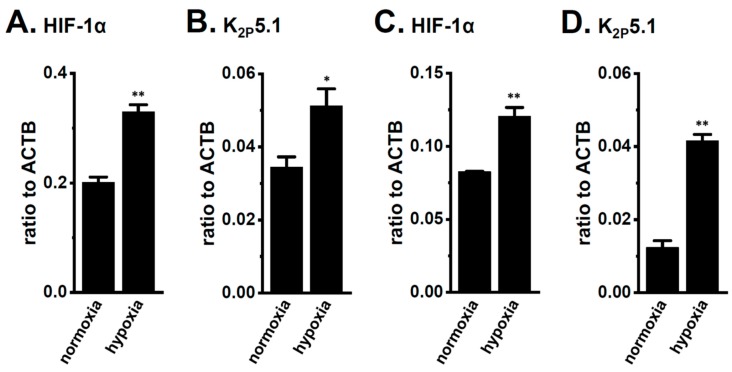
Up-regulation of HIF-1α and K_2P_5.1 by hypoxia (1.5% O_2_) for 24 h in the stimulated thymocytes of mice and the mouse T-cell line mCTLL-2. Real-time PCR assay for HIF-1α (**A**,**C**) and K_2P_5.1 (**B**,**D**) in stimulated thymocytes (**A**,**B**) and mCTLL-2 cells (**C**,**D**) exposed to hypoxic conditions (*n* = 4). Expression levels are shown as a ratio to ACTB. Results are expressed as means ± SEM. *, **: *p* < 0.05, 0.01 vs. normoxia.

**Figure 5:**
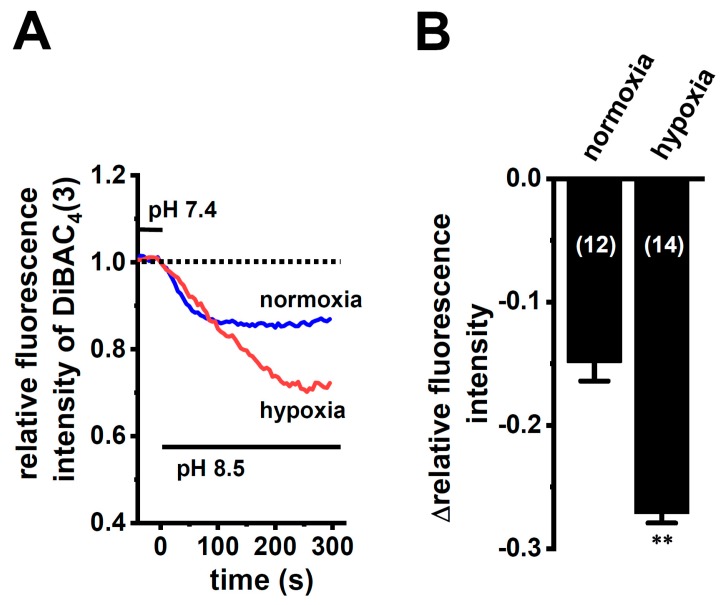
Enhanced K_2P_5.1 activity by hypoxia for 24 h in mCTLL-2 cells. (**A**) Time course of voltage-sensitive fluorescent dye imaging of alkaline-pH (pH 8.5)-induced hyperpolarizing responses in normoxia- and hypoxia-exposed mCTLL-2 cells. The fluorescent intensity of DiBAC_4_(3) before the change in pH from 7.4 to 8.5 (at 0 s) is expressed as 1.0. Images were measured every 5 s. (**B**) Summarized results of voltage-sensitive fluorescent dye imaging of alkaline-pH-induced hyperpolarizing responses in normoxia- and hypoxia-exposed mCTLL-2 cells. Cell numbers used in experiments are shown in parentheses. Results are expressed as means ± SEM. **: *p* < 0.01 vs. normoxia.

In order to show the hypoxia-exposed up-regulation of K_2P_5.1 proteins in stimulated CD4^+^ T cells, we then performed a functional analysis of K_2P_5.1 activity. As reported in our previous studies [9,29], K_2P_5.1 activity in mouse splenic CD4^+^ T cells can be estimated as alkaline pH-induced hyperpolarizing responses [a decrease in the fluorescence intensity of the voltage-sensitive fluorescent dye, bis-(1,3-dibutylbarbituric acid)trimethine oxonol, DiBAC_4_(3)]. Corresponding to greater increases in K_2P_5.1 expression under hypoxic conditions, the alkaline pH (pH 8.5 from pH 7.4)-induced hyperpolarizing responses were significantly larger in the hypoxia-exposed CD4^+^ T cells than in the normoxia-exposed cells (*p* = 0.0350) (Figure 3A,B). A pretreatment with clofilium (5 µM), a non-selective but potent K_2P_5.1 blocker, prevented alkaline pH-induced hyperpolarizing responses (Figure S2), as reported in a previous study [9]. A similar up-regulation of K_2P_5.1 with an increase in the expression of HIF-1α under hypoxic conditions was observed in Con-A-stimulated mouse thymocytes (*p* = 0.0005 and *p* = 0.0170 in HIF-1α and K_2P_5.1, respectively) (Figure 4A,B), as well as the K_2P_5.1-expressing mouse T-lymphocyte cell line, mCTLL-2 cells (*p* = 0.0084 and *p* = 0.0000 for HIF-1α and K_2P_5.1, respectively) (Figure 4C,D). Correspondingly, alkaline pH-induced hyperpolarizing responses were significantly larger in the hypoxia-induced mCTLL-2 cells (Figure 5A,B) than in the normoxia-exposed cells (*p* = 0.0000).

Recent studies have shown that hypoxia-induced alternative splicing events in cancerous cells [30,31]. We identified an *N* terminus-lacking, dominant-negative, spliced isoform of K_2P_5.1 in lymphoid cells [32], and we also found that a pre-mRNA inhibitor, pladienolide B, induced the down-regulation of functional, full-length K_2P_5.1 and the up-regulation of the non-functional spliced isoform (transcript variant X1) lacking three transmembrane domains and the first pore domain [29]. In the hypoxia-exposed CD4^+^ T cells, a full-length K_2P_5.1 with a molecular weight of approximately 1.7 kbp, was identified by DNA sequencing, but the spliced isoforms of K_2P_5.1 with molecular weights of less than 1 kbp were not. These results suggest that the dysregulated splicing of K_2P_5.1 is not involved in inflammation-associated hypoxia-induced enhancements in K_2P_5.1 activity in IBD model mice.

### 2.3. Effects of the HIF Inhibitor on K_2P_5.1 Expression and Activity in Stimulated Splenic CD4^+^ T Cells

**Figure 6:**
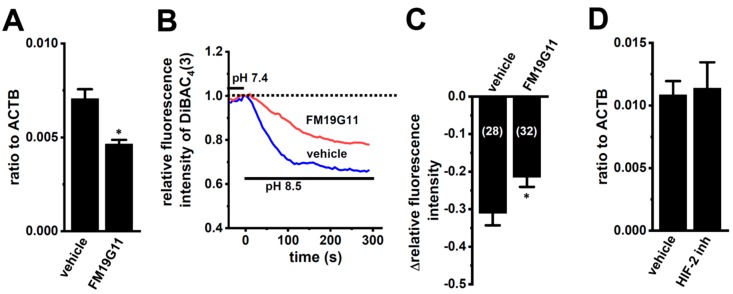
Decreased K_2P_5.1 expression level and activity by the pharmacological inhibition of HIF in hypoxia-exposed splenic CD4^+^ T cells. (**A**,**D**) Real-time PCR assay for K_2P_5.1 in hypoxia-exposed splenic CD4^+^ T cells that were treated with a vehicle, FM19G11 (1 µM) (**A**), and HIF-2 antagonist 2 (HIF-2 inh.) (10 µM) (**D**) for 24 h (*n* = 4). Expression levels are shown as a ratio to ACTB. (**B**) Voltage-sensitive fluorescent dye imaging of alkaline pH (pH 8.5)-induced hyperpolarizing responses in the vehicle- and FM19G11-treated groups. The fluorescent intensity of DiBAC_4_(3) before the change in pH from 7.4 to 8.5 at 0 s is expressed as 1.0. Images were measured every 5 s. (**C**) Summarized results of alkaline pH-induced hyperpolarizing responses in the vehicle- and FM19G11-treated groups. Cells were isolated from four different mice in each group. Cell numbers used in experiments are shown in parentheses. The values for fluorescent intensity were obtained by measuring the average for 1 min (12 images). Results are expressed as means ± SEM. *: *p* < 0.05 vs. vehicle control.

We examined the effects of the treatment with 1 µM of FM19G11, an HIF inhibitor, for 24 h on K_2P_5.1 expression and activity in hypoxia-exposed splenic CD4^+^ T cells. As shown in Figure 6A, K_2P_5.1 transcripts were significantly decreased by the FM19G11 treatment (*n* = 4, *p* = 0.0127). Correspondingly, alkaline pH (pH 8.5)-induced hyperpolarizing responses were reduced by the FM19G11 treatment (*p* = 0.0238) (Figure 6B,C). On the other hand, no significant changes in the K_2P_5.1 expression (*p* = 0.8294) were found by the treatment with a selective HIF-2 inhibitor, HIF-2 antagonist 2 (HIF-2 inh, 10 µM) [33] (*n* = 4) (Figure 6D). These results suggest that K_2P_5.1 transcription is regulated by HIF-1 in T cells.

### 2.4. Effects of the Class III Histone Deacetylase SIRT1 Inhibitor NCO-01 on K_2P_5.1 Expression and Activity in Stimulated CD4^+^ Cells

**Figure 7:**
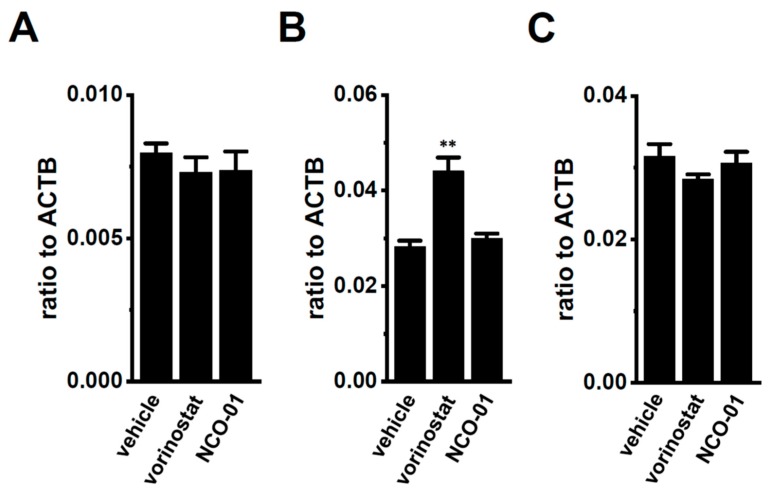
No significant changes in K_2P_5.1 expression by the pharmacological inhibition of sirtuin-1 (SIRT1) in the splenic CD4^+^ T cells of IBD model mice and the T-cell lineage. (**A**–**C**) Real-time PCR assay for K_2P_5.1 in the splenic CD4^+^ T cells of IBD model mice (**A**), stimulated thymocytes (**B**), and mCTLL-2 cells (**C**), all of which were treated with a vehicle, vorinostat (1 µM), and NCO-01 (50 µM) for 24 h [*n* = 4 mice (**A**,**B**) and *n* = 4 batches (**C**)]. Expression levels are shown as a ratio to ACTB. Results are expressed as means ± SEM. **: *p* < 0.01 vs. vehicle control.

Our previous findings indicated that the up-regulation of class I HDACs (HDAC2 and HDAC3) contributed to the increased expression and activity of K_Ca_3.1 in the CD4^+^ cells of the IBD model mice; however, class I HDAC inhibitor-induced decreases in the expression levels of K_2P_5.1 transcripts were not found [13]. Recent studies have revealed down-regulated sirtuin-1 (SIRT1) in IBD patients and model mice [27] and reduced SIRT1 activity by the down-regulation of NAD^+^ [34,35]. No significant changes were observed in the expression levels of K_2P_5.1 transcripts (*n* = 4, *p* = 0.6124) (Figure 7A) in the splenic CD4^+^ T cells of IBD model mice, treated with 1 µM of vorinostat, a pan-HDAC inhibitor, and 50 µM of NCO-01, an SIRT1/2 inhibitor for 24 h. NCO-01 inhibits SIRT1 activity with a half maximal inhibitory concentration value of approximately 50 μM [36]. The trypan blue dye exclusion test indicated 72.6 ± 6.4% and 70.5 ± 2.7% viable cells 24 h after the vehicle and NCO-01 treatments, respectively (*p* = 0.7376). Similarly, the expression level of K_2P_5.1 transcripts was not affected by the treatment with NCO-01 in stimulated thymocytes (*n* = 4, *p* > 0.05) (Figure 7B) or mCTLL-2 cells (*n* = 4, *p* > 0.05) (Figure 7C).

### 2.5. No Acute Compensatory Responses of K_2P_3.1 to K_2P_5.1 Up-Regulation in CD4^+^ T Cells of IBD Model Mice and Hypoxia-Exposed T Cells

**Figure 8:**
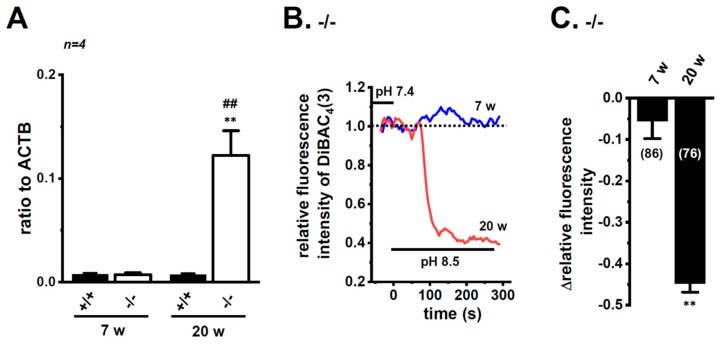
Compensatory increase in K_2P_3.1 expression and activity in the splenic CD4^+^ T cells of adult K_2P_5.1 homozygous knockout (KO) mice. (**A**) Real-time PCR assay for K_2P_3.1 in the splenic CD4^+^ T cells of wild-type (K_2P_5.1^+/+^) and K_2P_5.1 homozygous KO (K_2P_5.1^−/−^) mice at 7 (young) and 20 (adult) weeks old (*n* = 4). Expression levels are shown as a ratio to ACTB. (**B**) Voltage-sensitive fluorescent dye imaging of alkaline pH (pH 8.5)-induced hyperpolarizing responses in the splenic CD4^+^ T cells of K_2P_5.1^−/−^ mice at 7 and 20 weeks. The fluorescent intensity of DiBAC_4_(3) before the change in pH from 7.4 to 8.5 (at 0 s) is expressed as 1.0. Images were measured every 5 s. (**C**) Summarized results of alkaline pH (pH 8.5)-induced hyperpolarizing responses in the splenic CD4^+^ T cells of K_2P_5.1^−/−^ mice at 7 and 20 weeks. Cells were isolated from three different mice in each group. Cell numbers used in experiments are shown in parentheses. Results are expressed as means ± SEM. **: *p* < 0.01 vs. 7 weeks, K_2P_5.1^−/−^, ^##^: *p* < 0.01 vs. 20 weeks, K_2P_5.1^+/+^.

**Figure 9:**
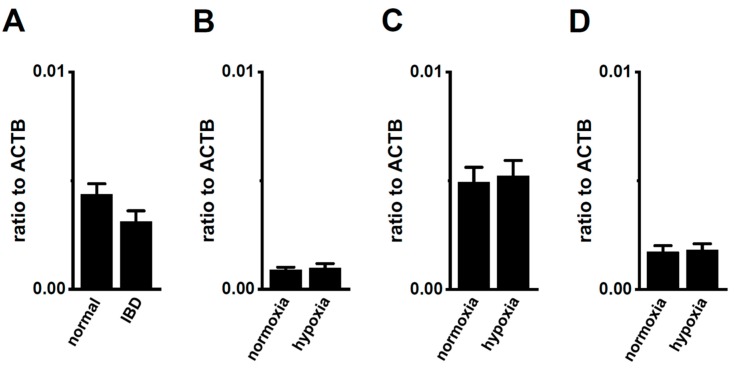
No compensatory changes in the expression levels of K_2P_3.1 transcripts in the splenic CD4^+^ T cells of IBD model mice or hypoxia-exposed T cells. (**A**–**D**) Real-time PCR assay for K_2P_3.1 in the splenic CD4^+^ T cells of ‘normal’ and ‘IBD’ model mice (**A**), hypoxia-exposed splenic CD4^+^ T cells (**B**), hypoxia-exposed thymocytes (**C**), and hypoxia-exposed mCTLL-2 cells (**D**) (*n* = 4 mice (**A**–**C**) and *n* = 4 batches (**D**)). Expression levels are shown as a ratio to ACTB. Results are expressed as means ± SEM.

Bittner et al. (2015) showed the compensatory up-regulation of K_2P_3.1 in K_2P_5.1-deficient mice [37]. In our previous study that used 7 week-old mice, the expression levels of K_2P_3.1 in the splenic CD4^+^ T cells of both the wild-type K_2P_5.1^+/+^ and homozygous K_2P_5.1 knockout K_2P_5.1^−/−^ mice were low, and alkaline pH (pH 8.5)-induced hyperpolarizing responses were not detected (Figure 8A,B). Further study revealed increases in K_2P_3.1 expression and activity in the splenic CD4^+^ T cells of K_2P_5.1^−/−^ mice older than 13 weeks. In the 20-week-old K_2P_5.1^−/−^ mice, marked increases were observed in K_2P_3.1 expression (*p* = 0.0001) (Figure 8A) and activity (*p* = 0.0000) (Figure 8B,C). The expression levels of other pH-sensitive K_2P_ channel subtypes, the K_2P_9.1 (TASK-3/KCNK9) and K_2P_16.1 (TALK-1/KCNK16) transcripts were very low (<0.001 in arbitrary units) in the CD4^+^ T cells of the K_2P_5.1^−/−^ mice, and no significant changes were observed between the K_2P_5.1^+/+^ and K_2P_5.1^−/−^ mice. In comparisons with the expression levels of K_2P_5.1, those of K_2P_3.1 were markedly lower in the CD4^+^ T cells, and no significant differences were found between the normal and IBD model mice (*n* = 4, *p* = 0.1252) (Figure 9A). Similar results were obtained in the hypoxia-exposed splenic CD4^+^ T cells (Figure 9B), thymocytes (Figure 9C), and mCTLL-2 cells (Figure 9D) (*n* = 4 mice (Figure 9B,C) and *n*= 4 batches (Figure 9D), *p* = 0.7393, 0.7799, and 0.9229, respectively). These results indicate that the alternations observed in K_2P_5.1 expression and activity in T cells in the present study were not accompanied by the compensation of K_2P_3.1.

## 3. Discussion

IFN-γ production was increased by the activation of the background K_2P_5.1 K^+^ channel function with its up-regulation in the CD4^+^ T cells of the IBD model mice, and most IBD symptoms were decreased in the K_2P_5.1 knockout mice [9]. Hypoxia is one of the crucial factors for an inflammatory microenvironment in autoimmune diseases, including IBD. Hypoxia-mediated signaling is involved in the pathogenesis of IBD by promoting an intestinal inflammatory response [17,18]. The main results of the present study are as follows: (1) Significant increases in HIF-1α were seen in the inflammatory CD4^+^ T cells of the IBD model mice that were isolated from a secondary lymphoid tissue, the spleen (Figure 1), (2) the HIF-1-mediated up-regulation of K_2P_5.1 was seen in stimulated CD4^+^ T cells (Figure 2, Figure 3, Figure 5 and Figure 6), and (3) no involvement of the class III HDAC, SIRT1, was in the post-translational modifications to K_2P_5.1 in these cells (Figure 7). Hypoxia-mediated up-regulation and the absence of SIRT1-mediated post-translational modifications to K_2P_5.1 were noted in other T-cell lineages (Figure 4, Figure 5 and Figure 7). Previous studies have shown that the dominant-negative spliced isoform of K_2P_5.1 prevents K_2P_5.1 activity by inhibiting the plasma membrane trafficking of dimeric channel assembly [29,38]. Under hypoxic conditions, no increase was noted in the expression level of the dominant-negative isoform of K_2P_5.1 in stimulated CD4^+^ T cells.

The alkaline pH-activated K_2P_ channel subfamily is composed of three members: K_2P_5.1/TASK2, K_2P_16.1/TALK2, and K_2P_17.1/TALK2, while the mouse homologue of K_2P_17.1 has not been molecularly identified. Furthermore, the acidic pH-sensitive K_2P_ channel subfamily, TASK (K_2P_3.1/TASK1 and K_2P_9.1/TASK3) also showed an increased channel activity with an alkaline pH stimulation. In stimulated CD4^+^ T cells, the expression of these K_2P_ subtypes, except for K_2P_5.1, was very weak, and no significant changes in their expression levels were observed following exposure to hypoxia. Similar to a recent study by Bittner et al. (2015) [37], K_2P_3.1 was up-regulated in the splenic CD4^+^ T cells of the ‘adult’ K_2P_5.1 homozygous knockout mice (Figure 8). The expression levels of K_2P_3.1 transcripts were markedly lower than those of K_2P_5.1 in the T-cell lineage, and these levels were not changed by the exposure to hypoxia (Figure 9). These results suggest that the alkaline pH-induced hyperpolarizing responses observed in the present study were mainly due to the activation of K_2P_5.1.

The transcriptional response to hypoxia is mainly mediated by epigenetic and post-translational modifications [39]. High levels of histone acetylation have been detected in biopsies from IBD patients and IBD model mice, and HDACs play an important role as regulators of inflammation. HDAC inhibitors decrease disease severity by suppressing inflammatory cytokine production in IBD model mice [40]. K_Ca_3.1, one of the major subtypes of K^+^ channels in T cells, was found to be up-regulated in the CD4^+^ T cells of IBD model mice [41]. We recently reported that K_Ca_3.1, but not K_2P_5.1, was regulated by the class I HDACs, HDAC2 and HDAC3 in activated CD4^+^ T cells [13]. Conversely, the HIF-1-downstream molecule SIRT1 was down-regulated in IBD patients and model mice [27]. As shown in Figure 7, the treatment with the SIRT1 inhibitor did not alter K_2P_5.1 transcription in the stimulated CD4^+^ T cells or the T-cell lineage. Therefore, in addition to class I HDACs, SIRT1 does not appear to be a post-translational regulator of K_2P_5.1 in the K_2P_5.1-overexpressing CD4^+^ T cells of IBD model mice.

The HIF-1 dimer binds to the target HIF-1-responsive element (HRE) region in order to activate target gene transcription [42]. Brazier et al. (2005) [43] cloned the K_2P_5.1 promoter, the activity of which is sensitive to oxygen, and the consensus binding sites for ETS (E-twenty six)-like 1 (Elk-1) were essential for transcriptional promotion by K_2P_5.1. Elk-1 may physically interact with HIF-1α. On the other hand, Cesari et al. (2004) showed that an Elk-1 deficiency did not induce changes in the proteomic displays of spleen extracts, and there were no immunological defects [44]. Shin et al. (2014) demonstrated that K_2P_5.1 was up-regulated under hypoxic conditions in an HIF-1α-dependent manner in B cells [24]. However, the underlying mechanisms of the HIF-1α-mediated up-regulation of K_2P_5.1 have not yet been elucidated. Under hypoxic conditions, the phosphorylation of STAT3 is increased in Th1 cells and leads to HIF-1α transcription [45]. Therefore, the hypoxia-induced activation of the STAT3 signaling pathway may be involved in the up-regulation of K_2P_5.1 in T cells. We examined the effects of treatments with STAT3 inhibitors (10 µM static, Abcam, Cambridge, UK) for 12 h on the expression levels of K_2P_5.1 transcripts in Con-A-stimulated splenic CD4^+^ T cells. However, the STAT3 inhibitor treatment caused cell death, and, thus, we were unable to measure the expression or activity of K_2P_5.1. This may be attributed to a defect in Con-A-induced T cell proliferation because the Con-A-induced phosphorylation of STAT3 promotes cell viability via the STAT3 signaling pathway [46]. The systematic evaluation of gene expression and DNA methylation in The Cancer Genome Atlas indicated the involvement of the hypomethylation of specific CpG loci with K_2P_5.1 overexpression in triple-negative breast cancer patients [47]. Genome- and epigenome-wide analyses of IBD [48,49] have systematically assessed the transcriptional and post-translational mechanisms underlying the up-regulation of K_2P_5.1 in T cells. K_2P_5.1 partners (such as 14–3–3 isoforms), stabilized protein expression, and promoted the plasma membrane trafficking of channel assembly [50]. Additionally, interactions between K_2P_5.1 and 14–3–3 may increase under hypoxic conditions. These findings suggest that increases in the interaction between 14–3–3 and K_2P_5.1 under inflammation-associated hypoxic conditions may be at least partly involved in enhancements in K_2P_5.1 activity in IBD.

The HIF-1α stimulation under inflammation-associated hypoxic conditions promotes CD4^+^ T cells to convert to regulatory T (T_reg_) cells, which produce the anti-inflammatory cytokine interleukin (IL)-10, and this plays an important role in protection against IBD [51]. The up-regulation of K_2P_5.1 with increases in HIF-1α expression was observed in the splenic CD4^+^CD25^+^ T cells of the IBD model mice (Figure S3A–C). These results suggest that K_2P_5.1 plays a role in the differentiation into T_reg_ cells and induction of IL-10. We previously reported that the Con-A stimulation converted splenic CD4^+^ T cells to CD4^+^CD25^high^Foxp3^high^ cells [29]. HIF-1α activates Foxp3 transcription in T_reg_ cells [51]. However, no significant changes were found in the expression levels of Foxp3 following the treatment with a potent but non-selective K_2P_5.1 blocker, clofilium, in Con-A-stimulated CD4^+^ T cells (*p* = 0.2566) (Figure S3D), thus suggesting that K_2P_5.1 activation is not involved in the differentiation into Foxp3^+^ T_reg_ cells. This issue is not the main subject of the present study, and, thus, further studies are needed to elucidate the physiological and pathophysiological roles of K_2P_5.1 channels in T_reg_ cells.

## 4. Materials and Methods

### 4.1. Preparation of the DSS-Induced Mouse Model of IBD and Isolation of CD4^+^ T Cells

Male C57 black 6 Jackson (C57BL/6J, 5–6 weeks of age) mice (Japan SLC, Shizuoka, Japan) were acclimatized for 1 week before the experiment. They were given drinking water containing 5% (*w*/*v*) DSS 5000 (Fujifilm Wako Pure Chemical, Osaka, Japan) ad libitum [9,13]. Like our previous study [9,13], male mice developed more significant and aggressive diseases than female ones [52]. Control mice were given drinking water only. Seven days after the administration of DSS, mice were euthanized, their spleens were isolated, and their symptoms like colitis and colonic inflammation were assessed, as described in our previous study [9,13]. All experiments were performed in accordance with the Guiding Principles for the Care and Use of Laboratory Animals in Nagoya City University (NCU) and Kyoto Pharmaceutical University (KPU) with the approval of the presidents (No. H29M-50, NCU; No. 16-12-091, KPU). K_2P_5.1 heterozygous knockout mice (K_2P_5.1^+/−^) that were bred in a C57BL6 background [B6; CB-Kcnk5Gt(pU-21)81Imeg] were purchased from the Center for Animal Resource and Development (CARD) (Kumamoto University, Kumamoto, Japan). Homozygous (K_2P_5.1^−/−^) knockout (KO) mice were generated by crossing K_2P_5.1^+/−^ males with K_2P_5.1^+/−^ females [9]. Genomic DNA isolation and RT-PCR were performed to distinguish the K_2P_5.1 wild type and gene-trapped KO mice, as described in our previous study [9].

Single-cell suspensions were prepared by pressing the spleen or thymus with a frosted grass slide and then filtering them through cell strainers. CD4^+^CD25^−^ or CD4^+^ T cells were isolated from cell suspensions by using the Dynabeads FlowComp mouse CD4^+^CD25^+^ or CD4^+^ kit according to the experimental manual supplied by Thermo Fisher Scientific (Waltham, MA, USA). Flow cytometric analyses confirmed that 95% of purified T cells were CD4^+^CD25^−^ or CD4^+^. Oxygen levels in culture media were adjusted with oxygen-absorbing packs (Mitsubishi Gas Chemical, Tokyo, Japan) and monitored by an oxygen monitor (Oxy-M, Jikco limited, Tokyo, Japan) by using the BIONIX hypoxic culture kit (Sugiyamagen, Tokyo, Japan).

### 4.2. Cell Culture

The mCTLL-2 was supplied by the RIKEN BioResource Center (RIKEN BRC) (Tsukuba, Japan). Cells were maintained at 37 °C, in 5% CO_2_ with an RPMI 1640 medium (FUJIFILM Wako Pure Chemical) supplemented with 10% fetal bovine serum (Sigma-Aldrich, St. Louis, MO, USA), a penicillin-streptomycin mixture (FUJIFILM Wako Pure Chemical), and 100 U/mL of IL-2 (Sigma-Aldrich).

### 4.3. RNA Extraction, Reverse Transcription (RT)-PCR, and Real-Time PCR

Total RNA extraction and RT-PCR from mouse splenic CD4^+^ T cells and mCTLL-2 cells were performed as previously reported [9,13]. The resulting complementary DNA (cDNA) products were amplified with gene-specific primers that were designed by Primer Express software (ver. 3.0.1, Thermo Fisher Scientific). Quantitative real-time PCR was performed by using Sybr Green Chemistry (SYBR Premix Ex Taq II) (TaKaRa BIO, Osaka, Japan) on the ABI 7500 Fast real-time PCR instrument (Thermo Fisher Scientific), as previously reported [9,13]. The following PCR primers for mouse clones were used for real-time PCR: K_2P_5.1 (GenBank accession number: NM_021542, 792–921), 130 bp; hypoxia-inducible factor (HIF)-1α (NM_010431, 963–1062), 100 bp; HIF-2α (NM_010137, 737–856), 120 bp; K_2P_3.1 (NM_010608, 692–812), 121 bp; K_2P_9.1 (NM_001033876, 757–877), 121 bp; K_2P_16.1 (NM_029006, 192–312), 121 bp; β-actin (ACTB) (NM_007393, 418–518), 101 bp. Regression analyses of the mean values of multiplex RT-PCRs for log_10_-diluted cDNA were used to generate standard curves. Unknown quantities relative to the standard curve for a particular set of primers were calculated, yielding the transcriptional quantitation of gene products relative to the endogenous standard, ACTB [9,13].

### 4.4. Molecular Cloning of K_2P_5.1 from Mouse Splenic CD4^+^ Cells

Gene-specific primers were designed based on the cloned mouse K_2P_5.1 (NM_021542, 281–1971, 1691 bp). The obtained PCR products were ligated into pcDNA3.1(+)/Neo^r^ (Thermo Fisher Scientific), and DNA sequences were obtained by using a custom DNA sequencing service (Eurofins Genomics, Tokyo, Japan).

### 4.5. Western Blotting

Protein lysates were prepared from mouse CD4^+^ T cells by using a lysis buffer for western blotting. After the quantification of protein concentrations by using the BIO-RAD DC^TM^ protein assay, protein lysates were subjected to SDS-PAGE (10%). Blots were incubated with anti-HIF-1α (28b) (Santa Cruz Biotechnology, Santa Cruz, CA, USA) and anti-ACTB (Sigma-Aldrich) antibodies, and they were then incubated with anti-mouse horseradish peroxidase-conjugated immunoglobulin G (IgG, Millipore, Temecula, CA, USA) [9,38]. An enhanced chemiluminescence detection system (Nacalai Tesque, Kyoto, Japan) was used to detect the bound antibody. The resulting images were analyzed by using Amersham Imager 600 (GE Healthcare Japan, Tokyo, Japan). The light intensities of the band signals relative to that of the ACTB signal were calculated by using ImageJ software (Ver. 1.42, NIH, Bethesda, MA, USA). In the summarized results, the relative protein expression levels in the control were expressed as 1.0.

### 4.6. Measurement of Membrane Potentials Using Fluorescent Voltage-Sensitive Dyes

Isolated splenic CD4^+^ T cells were cultivated in an RPMI 1640 medium that was supplemented with 10% heat-inactivated fetal calf serum (Merck, Darmstadt, Germany), antibiotics (penicillin and streptomycin mixture, Fujifilm Wako Pure Chemicals), Con-A (5 µg/mL), and IL-2 (10 U/mL) for 48 h. Membrane potentials were measured by using the voltage-sensitive dye DiBAC_4_(3), as previously reported [9,38]. Changes induced in the fluorescent intensity of DiBAC_4_(3) by alkaline pH (pH 8.5) were measured with an ORCA-Flash2.8 digital camera (Hamamatsu Photonics, Hamamatsu, Japan). Data collection and analyses were performed by using an HCImage system (Hamamatsu Photonics). Images were obtained every 5 s.

### 4.7. Chemicals

The sources of pharmacological agents were as follows: DiBAC_4_(3) (Dojindo, Kumamoto, Japan), clofilium (Sigma-Aldrich), Con-A (Sigma-Aldrich), FM19G11 (Sigma-Aldrich), and HIF-2 antagonist 2 (SML0883, Sigma-Aldrich). The SIRT1/2 inhibitor NCO-01 was supplied by Drs. Suzuki and Elboray. All other agents were obtained from Sigma-Aldrich, Nacalai Tesque, and FUJIFILM Wako Pure Chemical.

### 4.8. Statistical Analysis

Statistical evaluation was performed with the statistical software XLSTAT (version 2013.1, Microsoft Japan, Tokyo, Japan). To determine the significance of differences between two groups and among multiple groups, the unpaired (except Figure 1D)/paired (Figure 1D alone) Student’s *t* tests with Welch’s correction or Tukey’s tests were used. Results with *p* value of less than 0.05 or 0.01 were considered to be significant. Data are presented as means ± SEM.

## 5. Conclusions

The present study demonstrated that 1) a hypoxic characteristic in inflamed colonic tissues generates HIF-1α activation in the splenic CD4^+^ T cells of IBD model mice, and 2) the K_2P_5.1 channel is an HIF-1α target gene in splenic CD4^+^ T cells. Similar results were obtained in other T-cell lineage that were exposed to hypoxia. The present results suggest that post-translational modifications by class I and III HDACs are not involved in the transcriptional regulation of K_2P_5.1 in CD4^+^ T cells. A clearer understanding of the molecular mechanisms underlying the pathophysiological significance of HIF-1α-mediated K_2P_5.1 regulation in IBD patients is needed, and this can be done by using genome-wide and epigenome-wide association studies on DNA methylation and microRNA. The functional roles of K_2P_5.1 in the other T-cell subsets, such as T_reg_ cells, B cells, and myeloid cells, that are involved in the pathogenesis of IBD will provide valuable insights into autoimmune diseases.

## Supplementary Materials

Supplementary materials can be found at https://www.mdpi.com/1422-0067/21/1/38/s1.
