# Supplementary material for: Possible Contribution of Inflammation-Associated Hypoxia to Increased K2P5.1 K+ Channel Expression in CD4+ T Cells of the Mouse Model for Inflammatory Bowel Disease
Source: Int J Mol Sci. 2019 Dec 19;21(1):38. doi: 10.3390/ijms21010038 (PMC6981474; doi:10.3390/ijms21010038)
Supplement: Supplementary file 1 [file ijms-21-00038-s001.pdf]

Possible Contribution of Inflammation-Associated Hypoxia to Increased $K_{2P}5.1$ K^+ Channel Expression in $CD4^+$ T cells of the Mouse Model for Inflammatory Bowel Disease

Kyoko Endo, Hiroaki Kito, Ryo Tanaka, Junko Kajikuri, Satoshi Tanaka, Elghareeb E. Elboray, Takayoshi Suzuki and Susumu Ohya

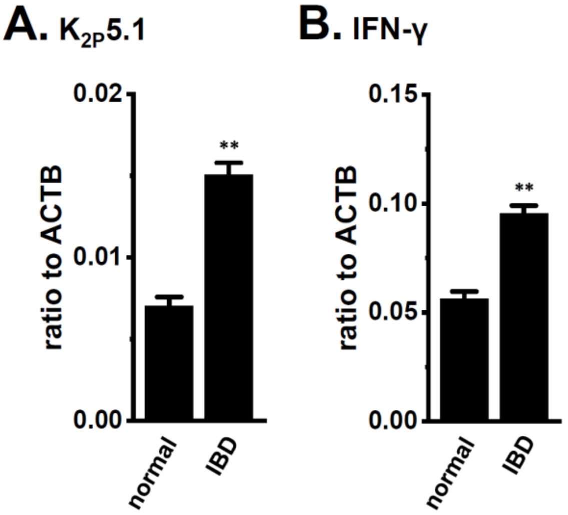

Figure S1. Increased expression levels of $K_{2P}5.1$ and IFN-γ transcripts in splenic $CD4^+$ T cells of IBD model mice. A, B: Real-time PCR assay for $K_{2P}5.1$ (A) and IFN-γ (B) in the splenic $CD4^+CD25^-$ T cells of 'normal' and 'IBD' model mice (n=4 mice for each). Expression levels were shown as a ratio to ACTB. Results are expressed as means ± SEM. **: $p < 0.01$ vs. normal.

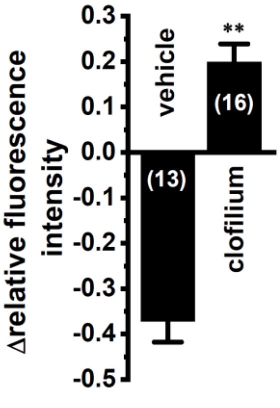

Figure S2. Disappearance of $K_{2P}5.1$ activity by application of clofilium (5 μM) in hypoxia-exposed splenic $CD4^+$ T cells. Summarized results of voltage-sensitive fluorescent dye imaging of alkaline pH (pH 8.5)-induced changes in relative fluorescent intensity of $DiBAC_4(3)$ in the presence ('clofilium') and absence ('vehicle') of 5 μM clofilium in hypoxia-exposed splenic $CD4^+$ T cells. Cells were isolated from two different mice in each group. Cell numbers used in experiments are shown in parentheses. Results are expressed as means ± SEM. **: $p < 0.01$ vs. vehicle control.

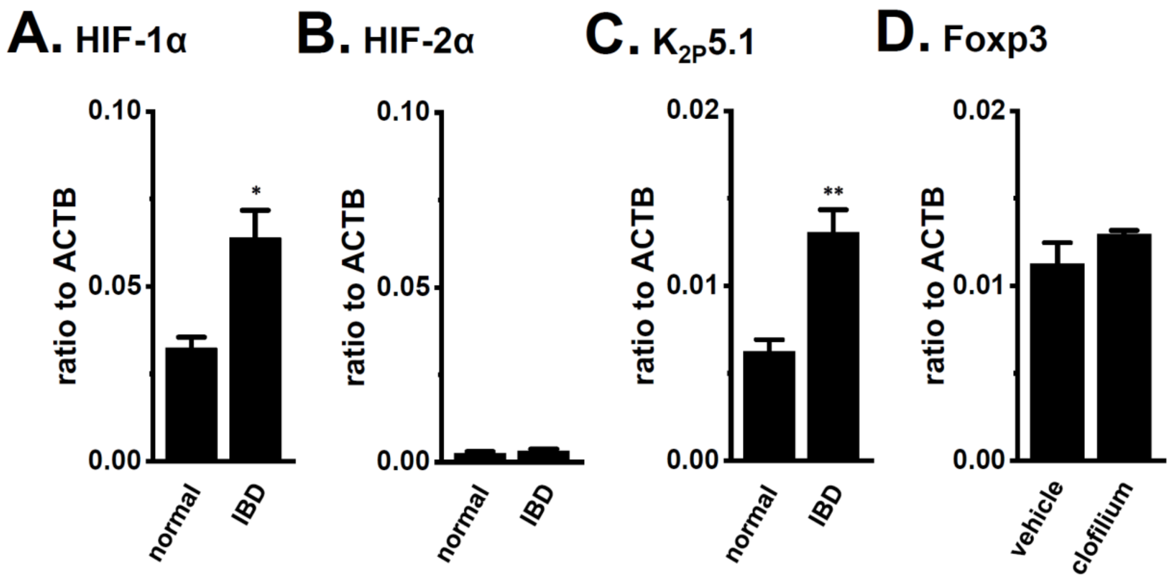

Figure S3. Expression levels of HIF-1α, HIF-2α, and K_{2P}5.1 in splenic $CD4^+$ $CD25^+$ T_{reg} cells of DSS-induced IBD model mice and effects of the treatment with 5 μM clofilium for 24 h on expression levels of Foxp3 in Con-A-stimulated splenic $CD4^+$ T cells. A-D: Real-time PCR assay for HIF-1α (A), HIF-2α (B), and K_{2P}5.1 (C) in the splenic $CD4^+CD25^+$ T_{reg} cells of 'normal' and 'IBD' model mice and Foxp3 (D) in clofilium-treated $CD4^+$ T cells (n=4 mice for each). Expression levels were expressed as a ratio to ACTB. Results are expressed as means ± SEM. *, **: $p < 0.05$, 0.01 vs. normal mice (normal) or vehicle control (vehicle).
